# Supplementary material for: FBW7 couples structural integrity with functional output of primary cilia
Source: Commun Biol. 2021 Sep 13;4:1066. doi: 10.1038/s42003-021-02504-4 (PMC8438042; doi:10.1038/s42003-021-02504-4)
Supplement: Supplementary file 2 — Supplementary Information [file 42003_2021_2504_MOESM2_ESM.pdf]

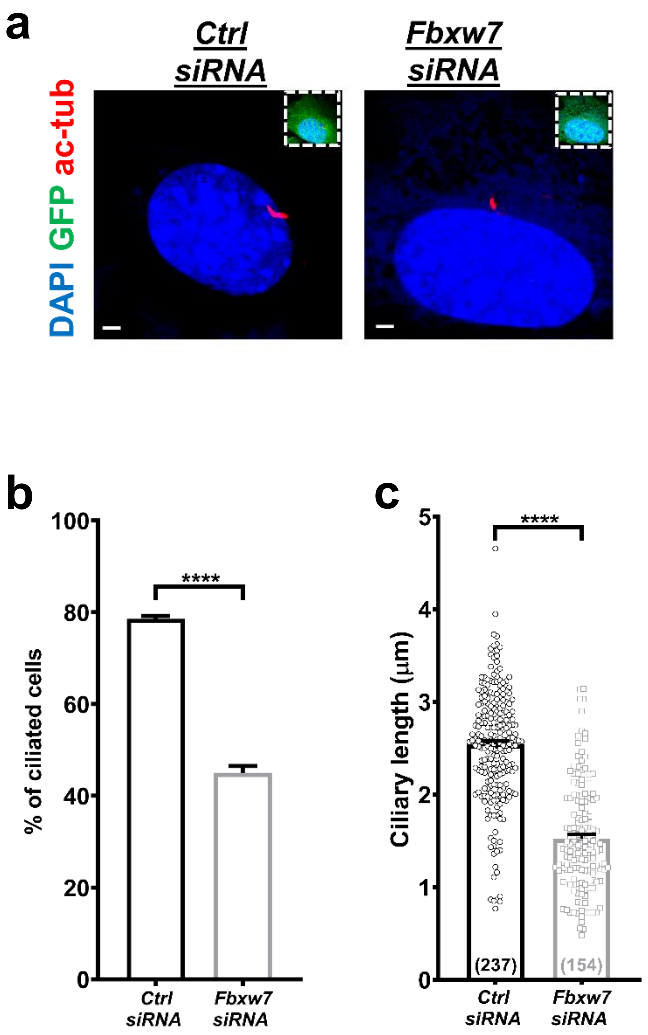

Supplementary Figure 1. Downregulation of *Fbxw7* reduces cilium incidence in C3H10T1/2 cells.

a) Representative images of C3H10T1/2 cells transfected with GFP and the indicated constructs, and serum starved for 24h. Scale bars: 2 µm.

b,c) Percent of ciliated cells (b) (n=3 experiments) and ciliary length (c) of cells in (a). For ciliary length analysis, the number of cells analyzed is indicated at the bottom of each bar in (c). Data are presented as means ± SEM. Student's t-test, ****$p < 0.0001$.

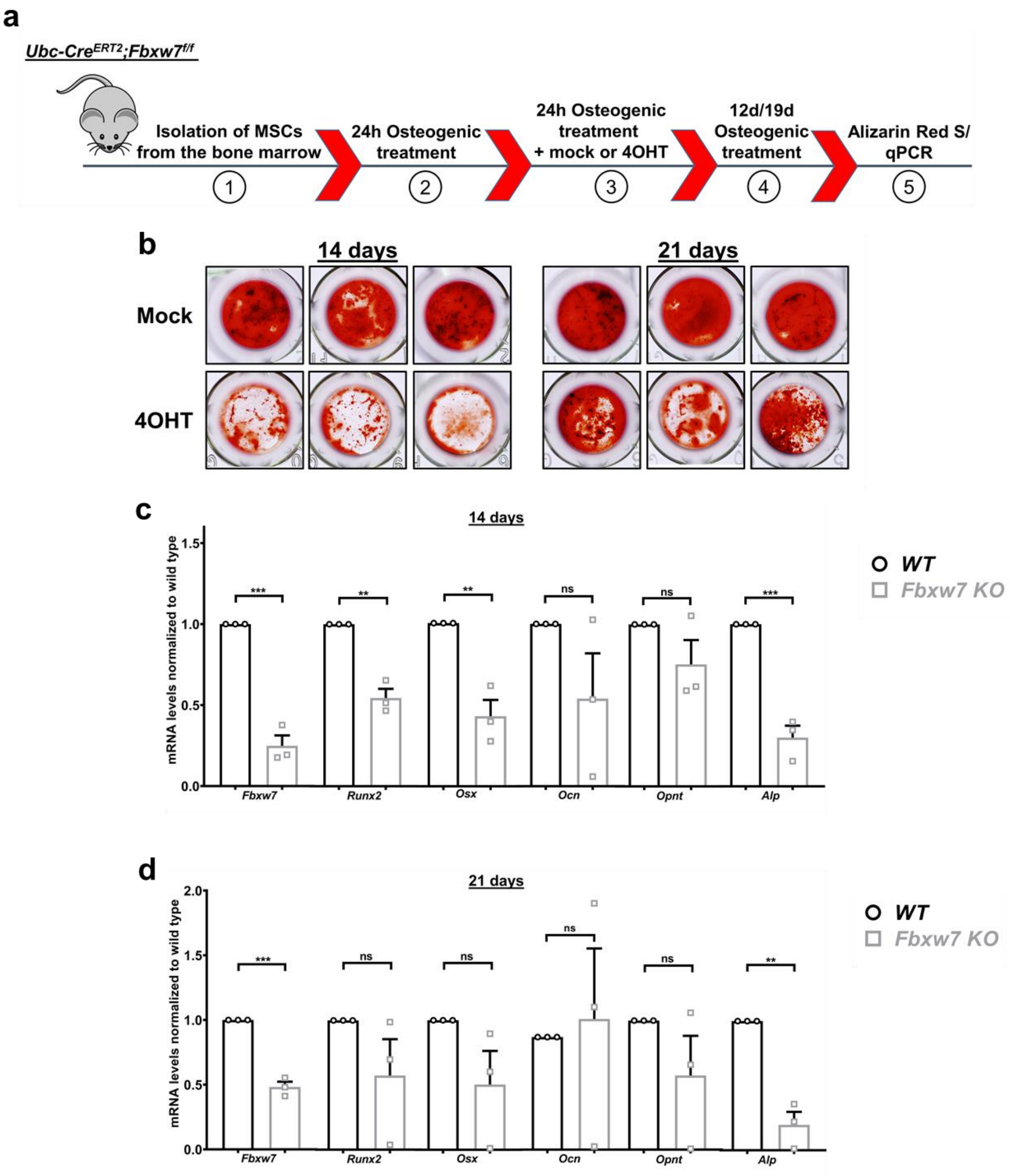

Supplementary Figure 2. *Fbxw7* deletion after osteogenic induction mildly suppresses differentiation of *UbcCre^{ERT2}; Fbxw7$^{f/f}$* MSCs to osteoblasts.

a) Diagram showing experimental setup for ex vivo deletion of *Fbxw7*. Isolation of MSCs from the bone marrow of *UbcCre^{ERT2}; Fbxw7$^{f/f}$* mice (1) was followed by 24h osteogenic treatment (2), 24h 4OHT-induced deletion of *Fbxw7* (3), continuation of osteogenic treatment (4) and analysis of osteoblast differentiation (5).
b-d) Osteoblast differentiation of *UbcCre^{ERT2}; Fbxw7$^{f/f}$*-derived MSCs treated with mock or 4OHT after initial osteogenic induction (n=3 different mice). Differentiation was measured at 14 and 21 days after osteogenic induction via Alizarin Red S staining (b) and mRNA levels of osteoblast differentiation markers *Runx2*, *Osterix* (*Osx*), *Osteocalcin* (Ocn), *Osteopontin* (*Opnt*) and Alkaline phosphatase (*Alp*) at 14 (c) and 21 days (d). mRNA levels of *Fbxw7* were analyzed to confirm deletion of the gene. Data are presented as means ± SEM. Student's t-test, **$p < 0.01$, ***$p < 0.001$, ****$p < 0.0001$. Images were taken from 96-well plates (a). Well diameter: 5 mm.

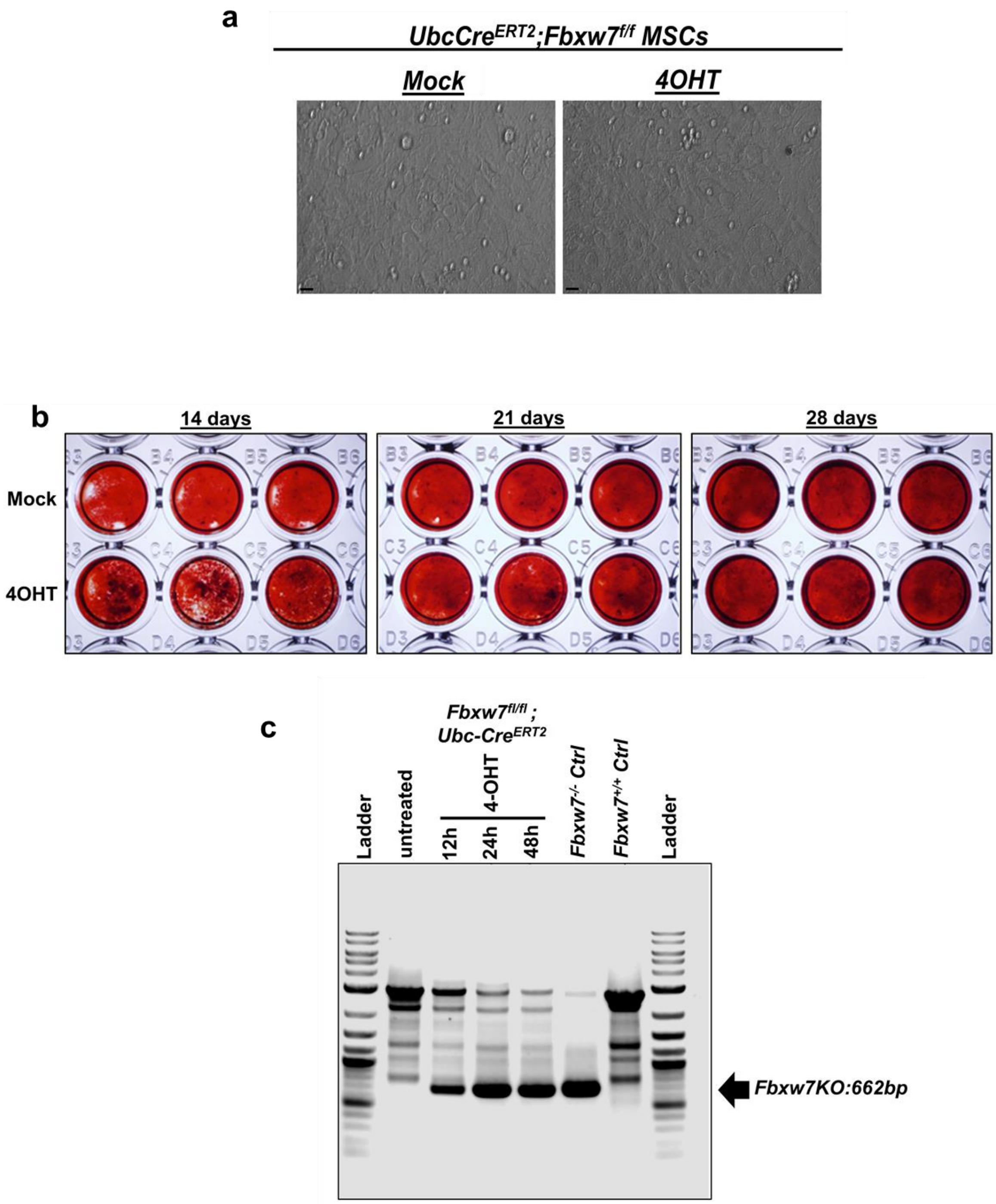

Supplementary Figure 3. No effect of 4OHT in osteoblast differentiation of wild type MSCs.

a) Representative DIC image of mock- or 4OHT- treated *UbcCre*^{ERT2}*; Fbxw7*$^{f/f}$ MSCs before the onset of differentiation induction. Scale bars: 15 µm.
b) Osteoblast differentiation of *Fbxw7*$^{f/f}$-derived MSCs treated with DMSO (mock) or 4OHT. Differentiation was measured at 14, 21 and 28 days via Alizarin Red S staining. Images were taken from 96-well plates: Well diameter: 5 mm.
c) Confirmation of deletion of *Fbxw7* in MSCs of *UbcCre*^{ERT2}*; Fbxw7*$^{f/f}$ after various duration of treatment with 4OHT. Black arrow indicates the PCR product after disruption of *Fbxw7*.

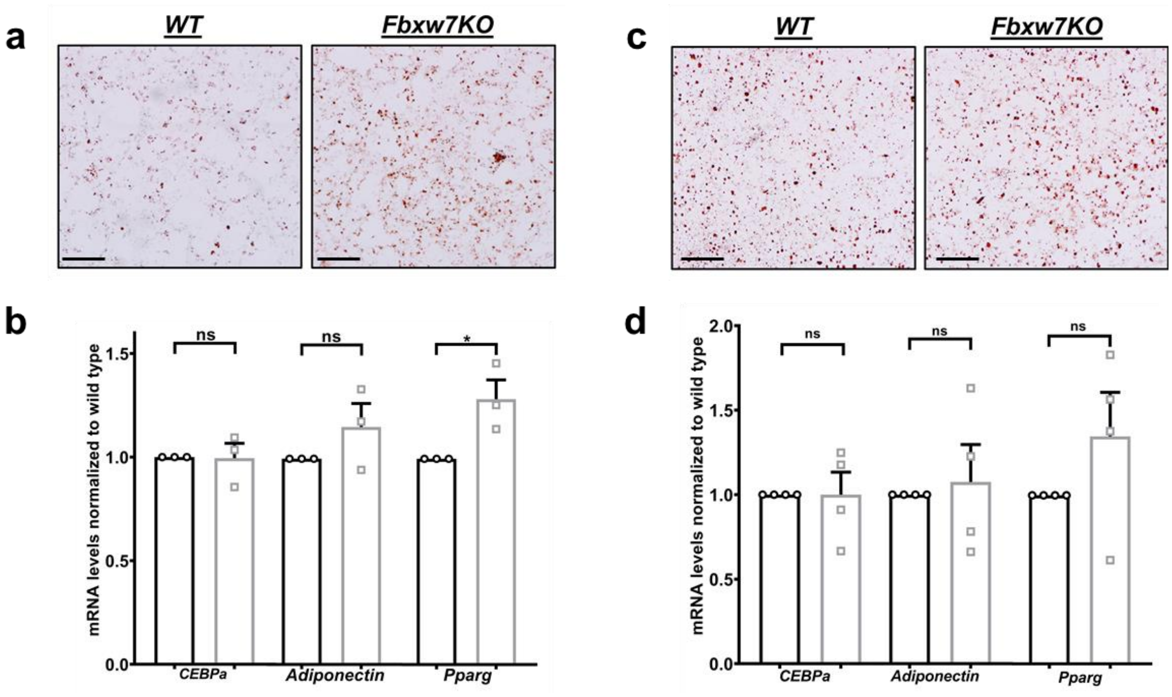

Supplementary Figure 4. Deletion of *Fbxw7* before adipogenic induction increases adipogenesis in *UbcCre^{ERT2}; Fbxw7$^{f/f}$* MSCs.

a-d) Adipogenic differentiation of *UbcCre^{ERT2}; Fbxw7$^{f/f}$*-derived MSCs treated with mock or 4OHT before (a,b) (n=3 different mice) or after initial adipogenic induction (c,d) (n=4 different mice). Differentiation was measured via Oil red O staining (a,c) and mRNA levels of adipogenic differentiation markers *CEBPa, Adiponectin* and *Pparγ* (b,d). Data are presented as means ± SEM. Student's t-test, *p < 0.05, **p < 0.01, ****p < 0.0001. Images were taken from a 96-well plate using 6.3x magnification (a). Scale bar: 500 μm.

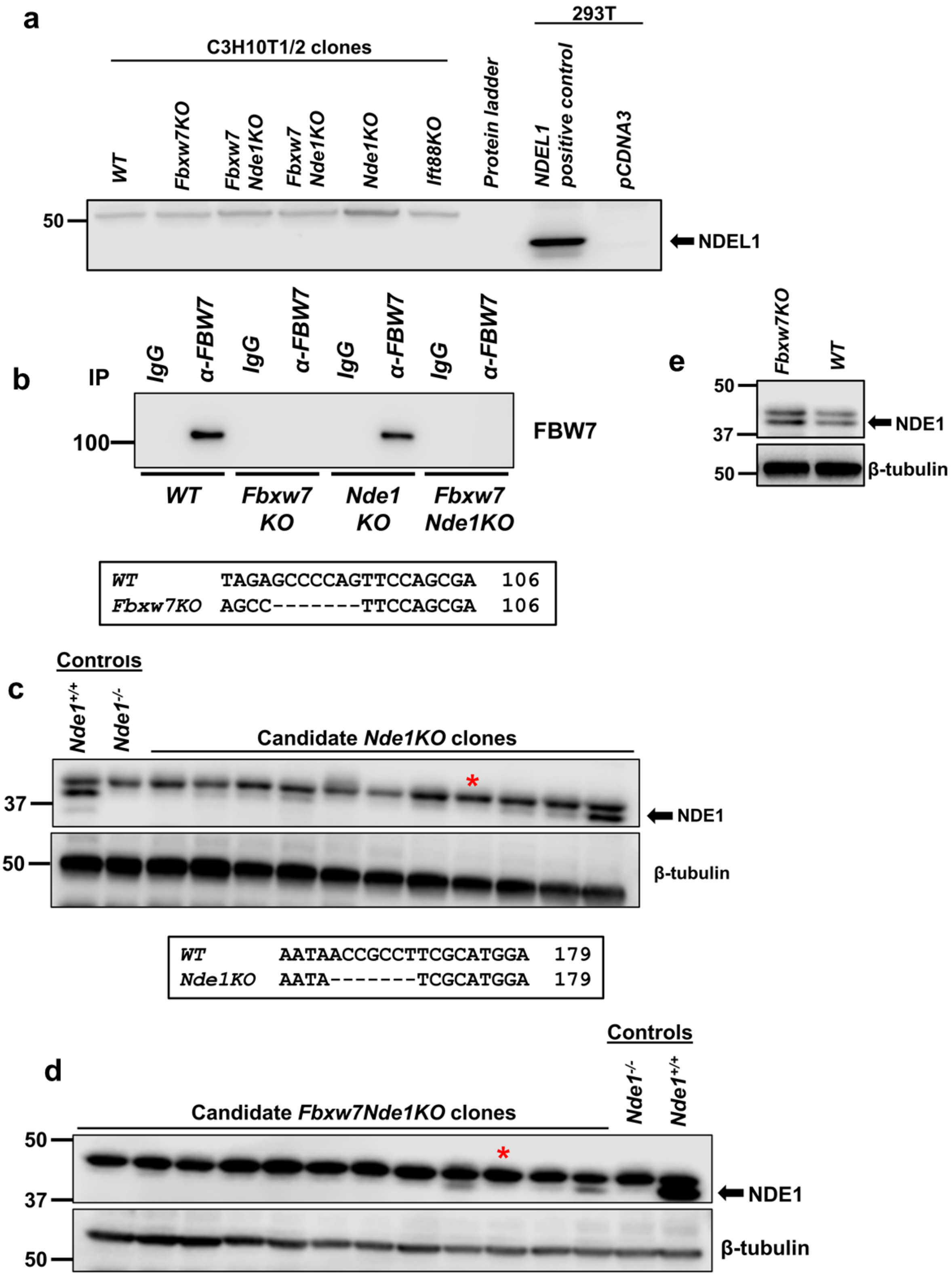
a
C3H10T1/2 clones
293T
WT
Fbxw7KO
Fbxw7 Nde1KO
Fbxw7 Nde1KO
Nde1KO
Ift88KO
Protein ladder
NDEL1 positive control
pCDNA3
50
NDEL1
b
IP
IgG
α-FBW7
IgG
α-FBW7
IgG
α-FBW7
IgG
α-FBW7
100
FBW7
WT
Fbxw7 KO
Nde1 KO
Fbxw7 Nde1KO
WT TAGAGCCCCAGTTCCAGCGA 106
Fbxw7KO AGCC-------TTCCAGCGA 106
e
Fbxw7KO
WT
50
37
NDE1
50
β-tubulin
c
Controls
Nde1+/+
Nde1-/-
Candidate Nde1KO clones
37
NDE1
50
β-tubulin
WT AATAACCGCCTTCGCATGGA 179
Nde1KO AATA-------TCGCATGGA 179
d
Controls
Nde1-/-
Nde1+/+
Candidate Fbxw7Nde1KO clones
50
37
NDE1
50
β-tubulin

Supplementary Figure 5. Expression of endogenous NDEL1 and validation of CRISPR/Cas9-mediated gene editing of indicated target genes in C3H10T1/2 cells.

a) Absence of NDEL1 expression in C3H10T1/2 clones. NDEL1 overexpression in HEK293T cells was used as a positive control.
b) Immunoprecipitation of endogenous FBW7 in wild type, *Fbxw7KO*, *Nde1KO* and *Fbxw7Nde1KO* C3H10T1/2 clones generated via CRISPR-Cas9 gene editing using *Fbxw7-* or *Nde1-* specific sgRNAs. Bottom: Sanger sequencing of exon 1 of mouse *Fbxw7* in *Fbxw7KO* C3H10T1/2 cells revealed a deletion around the Cas9 cleavage site.
c) Expression levels of endogenous NDE1 in various candidate *Nde1KO* clones. *Nde1*$^{+/+}$ and *Nde1*$^{-/-}$ MEF controls are shown in lanes 1 and 2, respectively, indicating that the lower band of the doublets is NDE1. Bottom: Sanger sequencing of the clone indicated with the red asterisk revealed a deletion around the Cas9 cleavage site.
d) Expression levels of endogenous NDE1 in various candidate *Fbxw7Nde1KO* clones. Previously generated *Fbxw7KO* C3H10T1/2 clone was transfected with *Nde1-* specific sgRNA to generate double *Fbxw7Nde1KO* C3H10T1/2 clones. *Nde1*$^{+/+}$ and *Nde1*$^{-/-}$ MEF controls are shown in lanes 13 and 14, respectively, indicating that the lower band in the doublets is NDE1.
e) Expression levels of endogenous NDE1 in the *Fbxw7KO* C3H10T1/2 clone under 24h serum starvation conditions. Arrow indicates the band corresponding to NDE1.

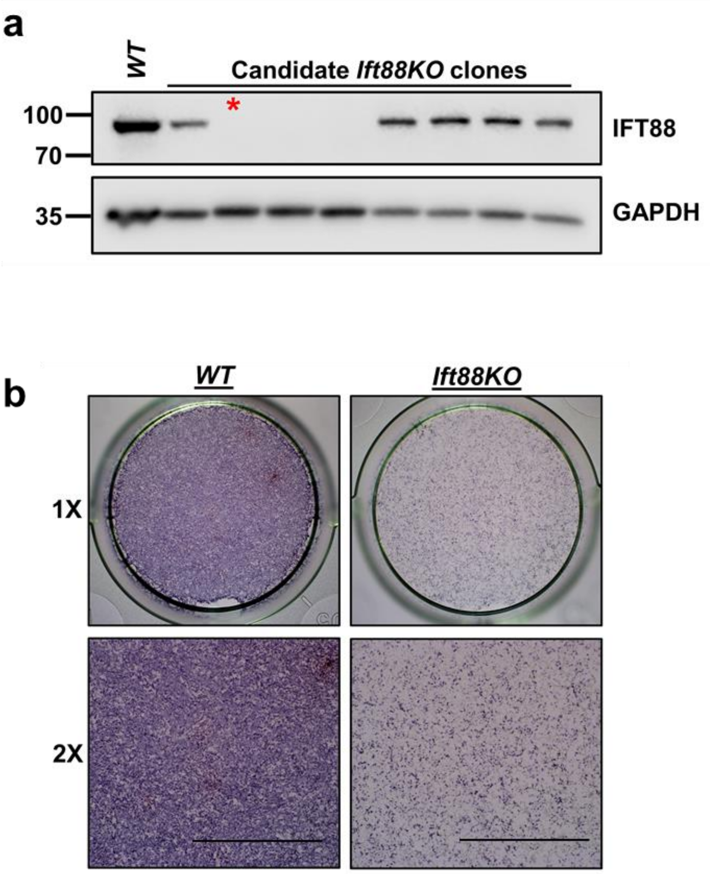

Supplementary Figure 6. Disruption of primary cilia compromises osteoblast differentiation in C3H10T1/2 cells.

a) Generation of *Ift88KO* C3H10T1/2 via CRISPR-Cas9 gene editing. Expression levels of endogenous IFT88. Red asterisk indicates null clone selected.
b) Wild type or the Ift88KO clone from (a) was induced via osteogenic medium and differentiation was analyzed via ALP staining. Images at 1x or 2x magnification were taken from 24-well plates. Well diameter: 16 mm. Scale bar: 5 mm.

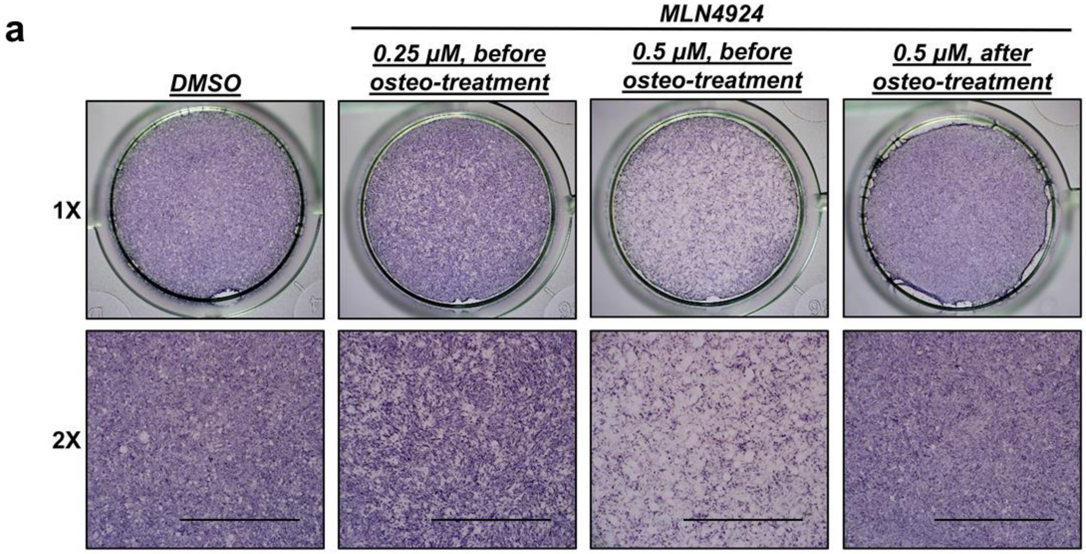

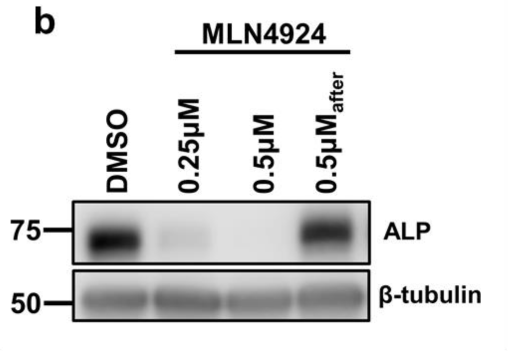

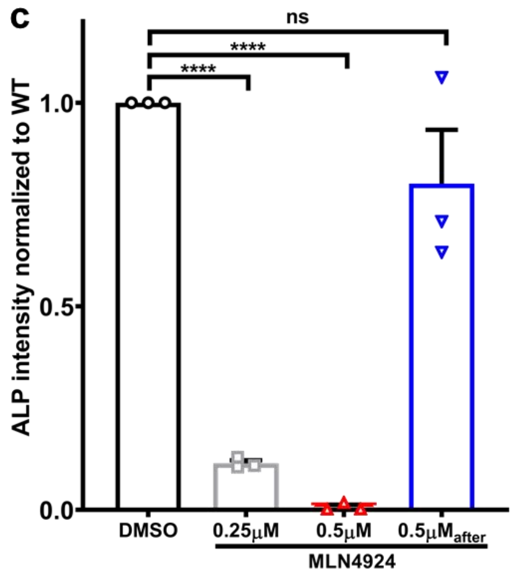

Supplementary Figure 7. Inhibition of FBW7 by MLN4924 before osteogenic induction severely compromises osteoblast differentiation in C3H10T1/2 cells.

a) ALP staining of wild type C3H10T1/2 cells after the indicated treatments. Images at 1x or 2x magnification were taken from 24-well plates. Well diameter: 16 mm. Scale bar: 5 mm.
b,c) Expression levels of ALP in wild type C3H10T1/2 cells after the indicated treatments (b) and summary data (c). Data are presented as means ± SEM. One-way ANOVA with Dunnett's multiple comparisons test, ****$p < 0.0001$.

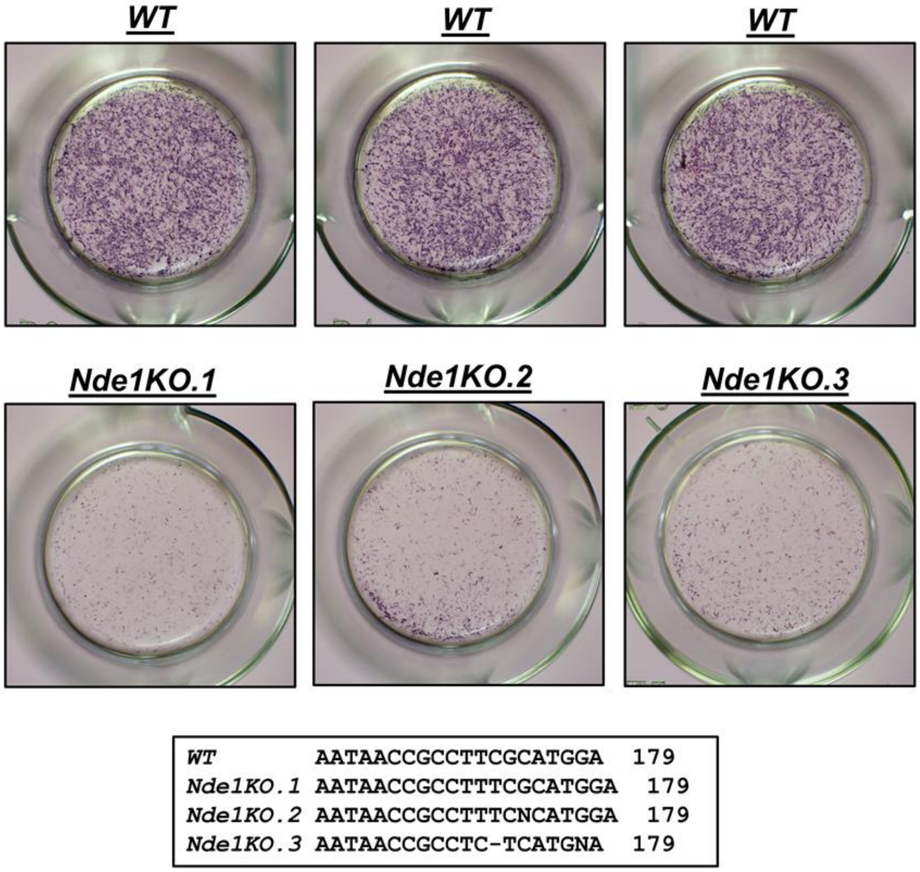

Supplementary Figure 8. Suppressed osteoblast differentiation in multiple *Nde1KO* C3H10T1/2 clones.

ALP staining of multiple Nde1KO C3H10T1/2 clones, compared to wild type. Bottom: Sanger sequencing of the clones revealed indels around the Cas9 cleavage site. Images were taken from 24-well plates. Well diameter: 16 mm.

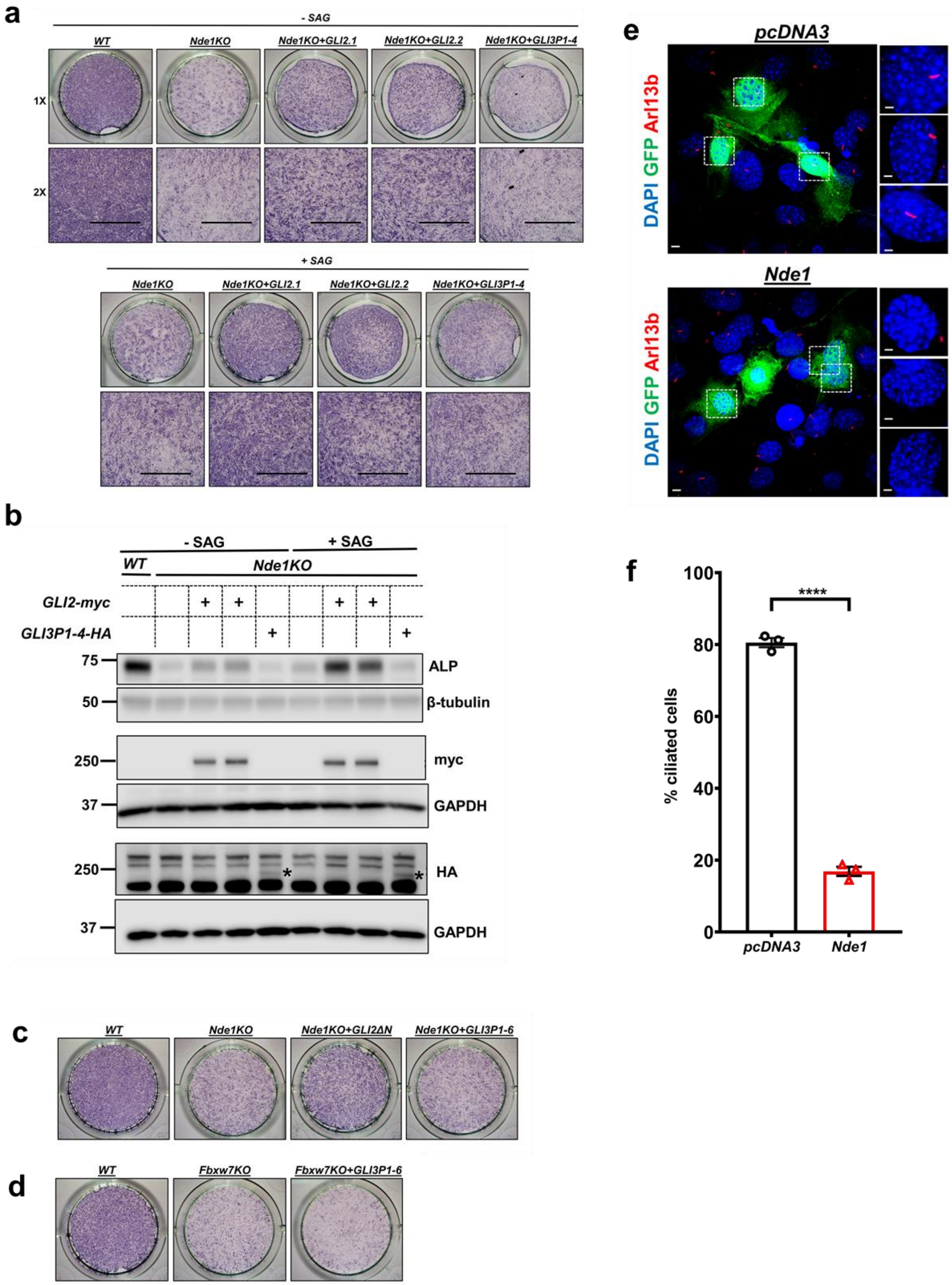

a
- SAG
WT
Nde1KO
Nde1KO+GLI2.1
Nde1KO+GLI2.2
Nde1KO+GLI3P1-4
1X
2X
+ SAG
b
- SAG
+ SAG
WT
Nde1KO
GLI2-myc
GLI3P1-4-HA
ALP
β-tubulin
myc
GAPDH
HA
c
Nde1KO+GLI2ΔN
Nde1KO+GLI3P1-6
d
Fbxw7KO
Fbxw7KO+GLI3P1-6
e
pcDNA3
Nde1
DAPI GFP Arl13b
f
% ciliated cells
****
pcDNA3
Nde1

Supplementary Figure 9. Modulation of the differentiation of *Nde1-KO* C3H10T1/2 cells by GLI2 and GLI3 and effect of NDE1 on cilium incidence of wild type C3H10T1/2 cells.

a,b) Representative ALP staining (a) and protein expression levels (b) of wild type and *Nde1KO* C3H10T1/2 cells transfected with the indicated constructs and treated with or without Smoothened agonist (SAG). Images at 1x or 2x magnification were taken from 24-well plates. Well diameter: 16 mm. Scale bar: 5 mm. (a) Asterisks indicate GLI3P1-4-HA band.
c) Representative ALP staining of wild type and *Nde1KO* C3H10T1/2 cells transfected with the indicated constructs. Well diameter: 16 mm.
d) Representative ALP staining of wild type and *Fbxw7KO* C3H10T1/2 cells transfected with the indicated constructs. Well diameter: 16 mm.
e,f) Representative images of wild type C3H10T1/2 cells transfected with GFP and the indicated constructs, and serum starved for 24h (e). Scale bars: 5 µm. Scale bars in insets: 2 µm. Percent of ciliated cells (f) (n=3 experiments) in (e). Data are presented as means ± SEM. Student's t-test, ****$p < 0.0001$.

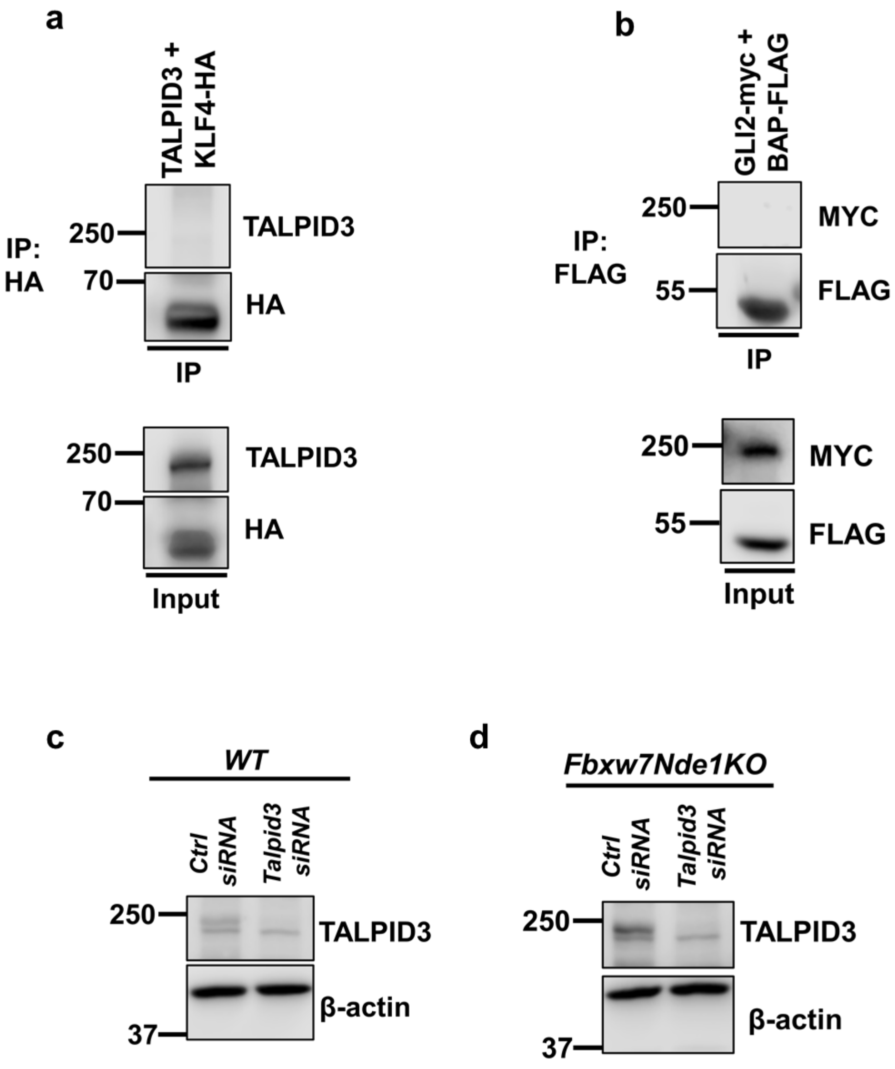

Supplementary Figure 10. No interaction of proteins of interest (TALPID3 or GLI2) with negative controls (KLF4 or BAP) and validation of silencing efficiency of *Talpid3*-specific siRNA.

a) No physical interaction of TALPID3 with HA-tagged KLF4 (KLF4-HA) in HEK293T cells.
b) No physical interaction of GLI2-myc with FLAG-tagged bacterial alkaline phosphate (BAP-FLAG) in HEK293T cells.
c,d) Expression levels of TALPID3 in wild type (f) or *Fbxw7Nde1KO* (g) C3H10T1/2 cells transfected with a *Talpid3* construct and mock or *Talpid3* specific siRNA

Suppl. Fig 5a

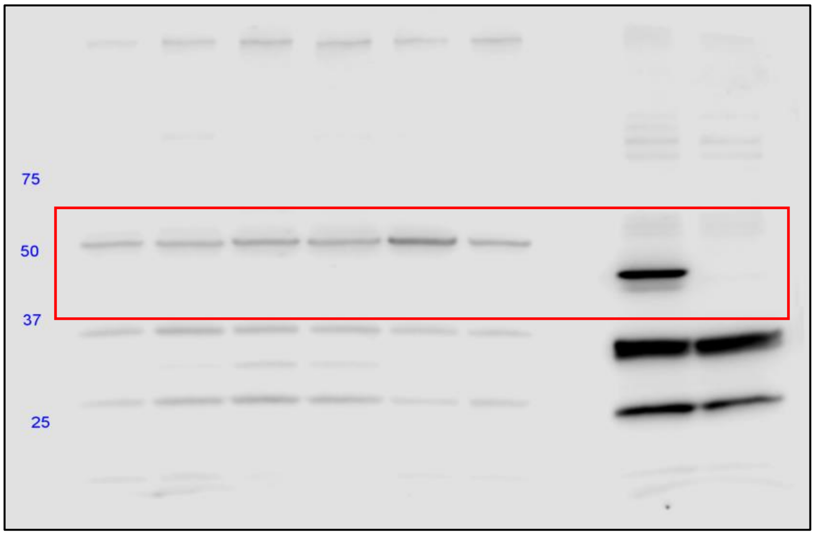

Suppl. Fig 5b

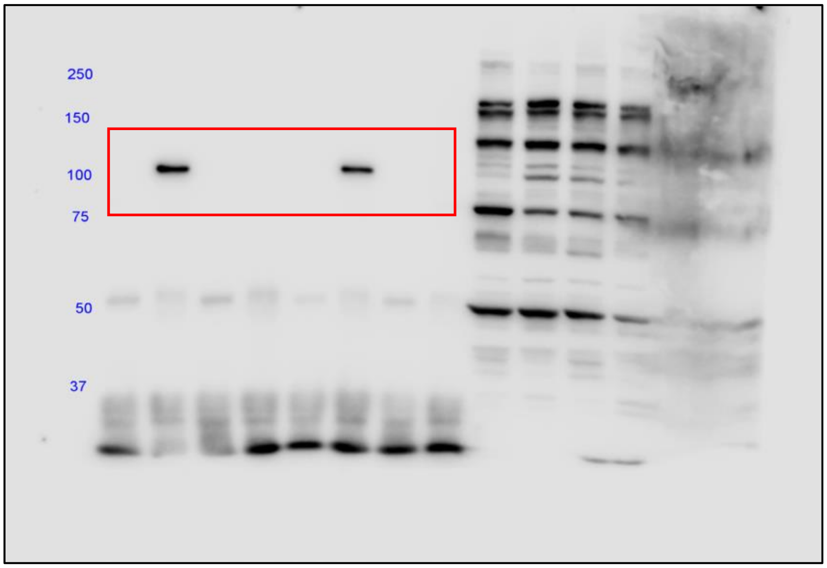

Suppl. Fig 5c

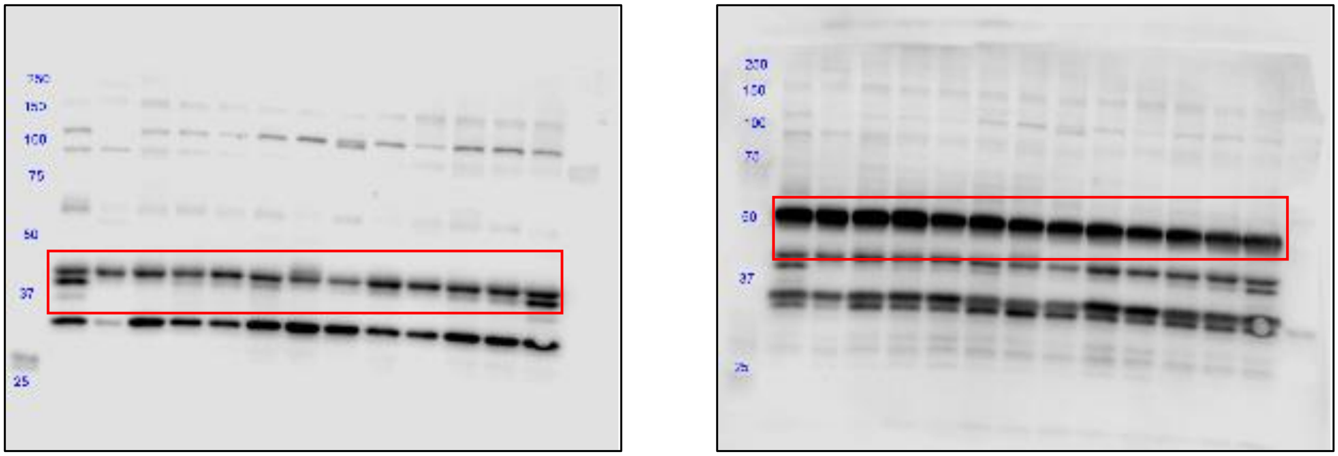

Suppl. Fig 5d

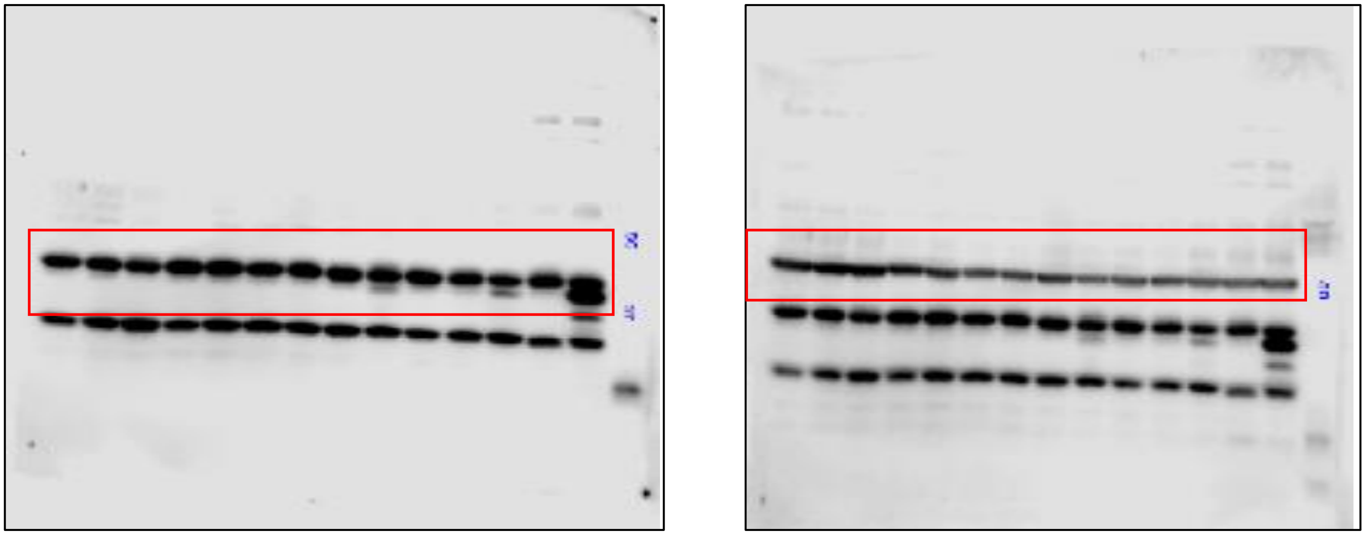

Suppl. Fig 5e

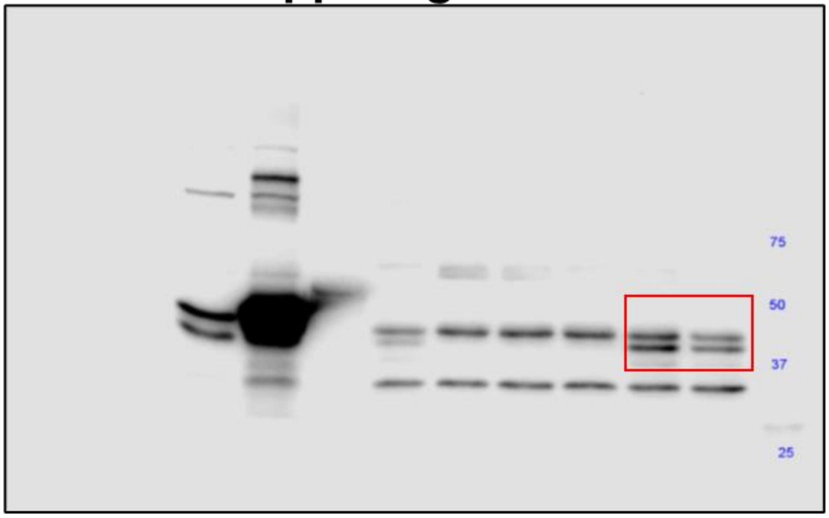

Suppl. Fig 5e

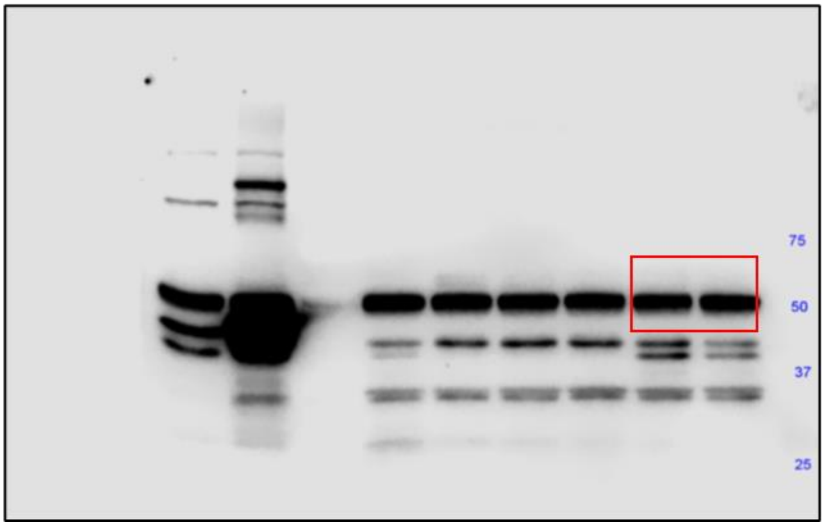

Suppl. Fig 10c **Suppl. Fig 10d**

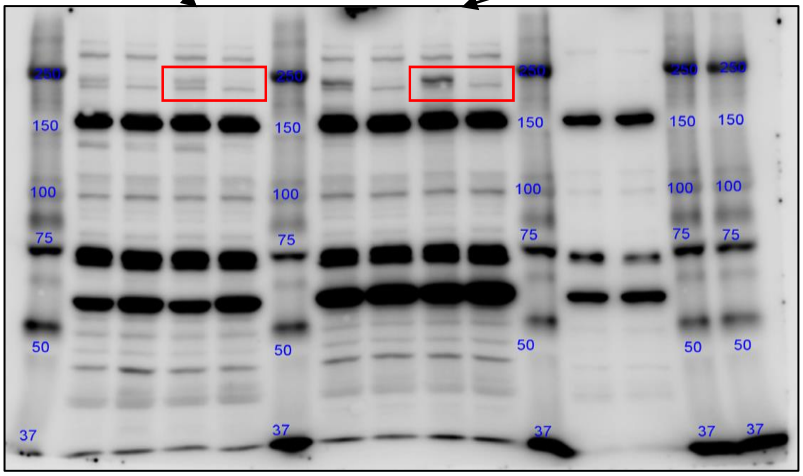

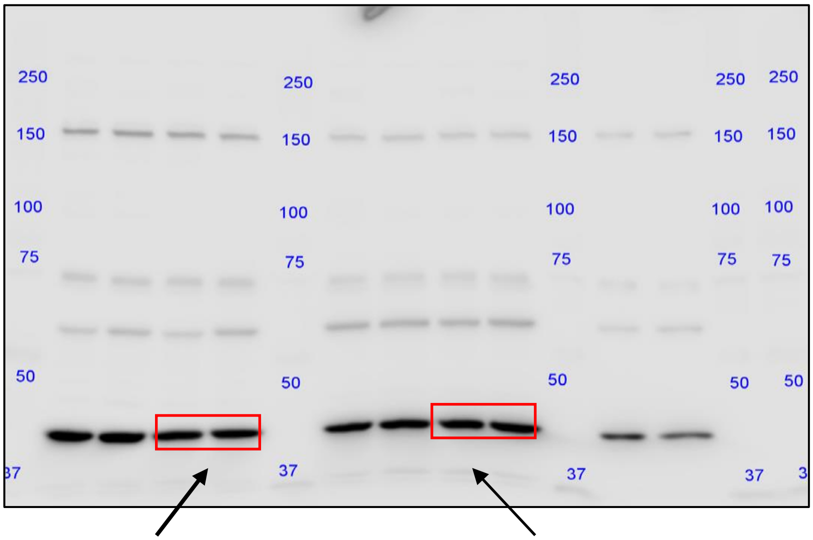

Suppl. Fig 10c **Suppl. Fig 10d**
