# Supplementary material for: FBW7 couples structural integrity with functional output of primary cilia
Source: Commun Biol. 2021 Sep 13;4:1066. doi: 10.1038/s42003-021-02504-4 (PMC8438042; doi:10.1038/s42003-021-02504-4)
Supplement: Supplementary file 5 — Reporting Summary [file 42003_2021_2504_MOESM5_ESM.pdf]

nature research

Corresponding author(s): Leonidas Tsiokas

Last updated by author(s): May 14, 2021

Reporting Summary

Nature Research wishes to improve the reproducibility of the work that we publish. This form provides structure for consistency and transparency in reporting. For further information on Nature Research policies, see our Editorial Policies and the Editorial Policy Checklist.

Statistics

For all statistical analyses, confirm that the following items are present in the figure legend, table legend, main text, or Methods section.

n/a	Confirmed	
☐	☒	The exact sample size (*n*) for each experimental group/condition, given as a discrete number and unit of measurement
☒	☐	A statement on whether measurements were taken from distinct samples or whether the same sample was measured repeatedly
☐	☒	The statistical test(s) used AND whether they are one- or two-sided<br>*Only common tests should be described solely by name; describe more complex techniques in the Methods section.*
☒	☐	A description of all covariates tested
☒	☐	A description of any assumptions or corrections, such as tests of normality and adjustment for multiple comparisons
☐	☒	A full description of the statistical parameters including central tendency (e.g. means) or other basic estimates (e.g. regression coefficient) AND variation (e.g. standard deviation) or associated estimates of uncertainty (e.g. confidence intervals)
☒	☐	For null hypothesis testing, the test statistic (e.g. F, t, r) with confidence intervals, effect sizes, degrees of freedom and P value noted<br>*Give P values as exact values whenever suitable.*
☒	☐	For Bayesian analysis, information on the choice of priors and Markov chain Monte Carlo settings
☒	☐	For hierarchical and complex designs, identification of the appropriate level for tests and full reporting of outcomes
☒	☐	Estimates of effect sizes (e.g. Cohen's d, Pearson's r), indicating how they were calculated

Our web collection on statistics for biologists contains articles on many of the points above.

Software and code

Policy information about availability of computer code

Data collection	No algorithms or software were used for data collection.
Data analysis	Data were analyzed via Graphpad Prism 8 Software. Statistical significance was determined by two-tailed Student's t-test or one-way ANOVA. For ANOVA, wherever means were compared only with the control group Dunnett's test was used, whereas in the case of comparisons of multiple pairs of means, Sidak's test was used.

For manuscripts utilizing custom algorithms or software that are central to the research but not yet described in published literature, software must be made available to editors and reviewers. We strongly encourage code deposition in a community repository (e.g. GitHub). See the Nature Research guidelines for submitting code & software for further information.

Data

Policy information about availability of data

All manuscripts must include a data availability statement. This statement should provide the following information, where applicable:

- Accession codes, unique identifiers, or web links for publicly available datasets
- A list of figures that have associated raw data
- A description of any restrictions on data availability

The data that support the findings of this study are available from the corresponding author upon reasonable request.

Field-specific reporting

Please select the one below that is the best fit for your research. If you are not sure, read the appropriate sections before making your selection.

- [x] Life sciences
- [ ] Behavioural & social sciences
- [ ] Ecological, evolutionary & environmental sciences

For a reference copy of the document with all sections, see nature.com/documents/nr-reporting-summary-flat.pdf

Life sciences study design

All studies must disclose on these points even when the disclosure is negative.

Sample size: No sample size determination was performed in the study. All experiments were performed at least three times in an unbiased manner.

Data exclusions: No data were excluded.

Replication: All experiments were replicated at least three times.

Randomization: Experimental setup was randomized.

Blinding: For cell culture experiments, investigators were not blinded to group allocation during data collection and/or analysis, however all experimental controls and samples were analyzed in the same manner.
Animal experiments (microcomputed analysis) were done in a blinded way.

Reporting for specific materials, systems and methods

We require information from authors about some types of materials, experimental systems and methods used in many studies. Here, indicate whether each material, system or method listed is relevant to your study. If you are not sure if a list item applies to your research, read the appropriate section before selecting a response.

Materials & experimental systems

n/a	Involved in the study
☐	☒ Antibodies
☐	☒ Eukaryotic cell lines
☒	☐ Palaeontology and archaeology
☐	☒ Animals and other organisms
☒	☐ Human research participants
☒	☐ Clinical data
☒	☐ Dual use research of concern

Methods

n/a	Involved in the study
☒	☐ ChIP-seq
☒	☐ Flow cytometry
☒	☐ MRI-based neuroimaging

Antibodies

Antibodies used:
Thermofisher : ALP (#PA5-47419)
Proteintech: NDE1 (#10233-1-AP), TALPID3 (#24421-1-AP), IFT88 (#13967-1-AP)
Santa Cruz: β-tubulin (#sc-9104), HA (#sc-7392), β-actin (#sc-4778)
Cell Signaling: Myc-tag (#2276),
Sigma: FLAG (#F1804), acetylated α-tubulin (#T6793)
Abcam: NDEL1 (#ab25959), CD106-FITC (#ab24853)
Genetex: GAPDH (#GTX82560)
Abnova: FBW7 (H00055294-B01P)
Secondary antibodies for Western blot: Mouse anti-goat IgG-HRP (Santa Cruz, #sc-2354), sheep anti-mouse IgG-HRP (Cytiva, #NA931), sheep anti-rabbit IgG-HRP (Cytiva, #NA934)
Secondary antibodies for immunofluorescence: goat anti-mouse Alexa594 (Thermofisher, #A11020) and donkey anti-goat (Thermofisher, #A11057).

Validation: Antibodies were previously validated by the manufacturer. NDE1 antibody was validated in the manuscript using mouse embryonic fibroblasts from Nde1-null mice.

Eukaryotic cell lines

Policy information about cell lines

Cell line source(s)	Cell lines were obtained from ATCC.
Authentication	No authentication was performed on cell lines.
Mycoplasma contamination	No mycoplasma contamination.
Commonly misidentified lines (See ICLAC register)	No commonly misidentified lines were used.

Animals and other organisms

Policy information about studies involving animals; ARRIVE guidelines recommended for reporting animal research

Laboratory animals	C57BL/6 mice were purchased from The Jackson Laboratory (Bar Harbor, ME, USA).
Wild animals	No wild animals were involved in the study.
Field-collected samples	No samples were collected from the field.
Ethics oversight	Animal experiments were performed according to protocols approved by the Institutional Animal Care and Use Committee (IACUC) of the University of Oklahoma Health Sciences Center.

Note that full information on the approval of the study protocol must also be provided in the manuscript.
